# Alcohol affects the emotional modulation of cognitive control: an event-related brain potential study

**DOI:** 10.1007/s00213-012-2664-6

**Published:** 2012-02-28

**Authors:** Anja S. Euser, Ingmar H. A. Franken

**Affiliations:** 1 Institute of Psychology, Erasmus University Rotterdam, Woudestein T12-59, P.O. Box 1738, 3000 DR Rotterdam, The Netherlands; 2 Department of Child and Adolescent Psychiatry, Erasmus Medical Center Rotterdam, Rotterdam, The Netherlands

**Keywords:** Alcohol, Emotion, Cognitive control, Event-related potentials (ERPs), N200, P300, Go/No-Go

## Abstract

**Objective:**

The present study aimed to determine whether alcohol affects the emotional modulation of cognitive control and its underlying neural mechanisms, which is pivotal to an understanding of the socially maladaptive behaviors frequently seen in alcohol-intoxicated individuals.

**Method:**

Event-related potentials (ERPs) were recorded in male participants receiving either a moderate dose of alcohol (0.65 g/kg alcohol; *n* = 32) or a non-alcoholic placebo beverage (*n* = 32) while performing an emotional Go/No-Go task that required response execution (Go trials) to pictures of a “target” emotional facial expression (angry, happy, neutral) and response inhibition (No-Go trials) to a different “non-target” expression.

**Results:**

Overall, N200 and P300 amplitudes were more enhanced during No-Go than Go trials. Interestingly, alcohol-intoxicated individuals displayed larger No-Go N200 amplitudes across all emotional conditions than controls, accompanied by decreased task performance (i.e., more errors), particularly in response to angry faces. P300 amplitude in the alcohol group was significantly reduced for both Go and No-Go trials, but only following angry and happy emotional expressions.

**Conclusions:**

These results suggest that alcohol-intoxicated individuals need to effortfully activate more cognitive resources during the early inhibition process in order to regulate a response than controls. Moreover, alcohol affected the emotional modulation of both response inhibition and execution in the later stages of cognitive control. Alcohol dampened emotional responsiveness, which may restrict the availability of attentional resources for cognitive control. Yet, these findings may underlie the lack of control in alcohol-intoxicated individuals when faced with emotionally or socially challenging situations.

When individuals are intoxicated by alcohol, they make more impulsive choices that disregard future consequences which eventually results in socially inappropriate behaviors, such as aggression, interpersonal violence, and unwarranted sexual advances (e.g., Heinz et al. 2011). One could argue that alcohol has a negative influence on adaptive behaviors. For adaptive behavior, it is essential to selectively respond to relevant environmental cues while, at the same time, inhibiting competing, spontaneous, but inappropriate reactions (Schulz et al. 2009). These abilities of appropriate response selection (i.e., response execution) and inhibition, which are known under the general term of cognitive control (Mostofsky & Simmonds, 2008; Posner & Dehaene, 1994), are particularly important in social and emotional contexts (e.g., Delplanque et al. 2004; Goldstein et al. 2007). In everyday life, people constantly experience emotionally evocative situations, and the ability to inhibit responses to irrelevant emotional cues is critical for social functioning (e.g., inhibiting inappropriate aggressive behavior in response to a perceived threatening emotional facial cue). Given the clear association between socially appropriate behavior and response inhibition in the face of emotional cues, examining whether and if so how emotions affect cognitive control processes (i.e., response execution and inhibition) may provide important insights into the socially maladaptive and disinhibited behaviors frequently seen in alcohol-intoxicated individuals. However, despite its suggested relevance to social functioning, the acute effects of alcohol on cognitive control processes are mainly studied in non-social domains.

The detrimental effects of alcohol on cognitive control processes, including its underlying neural mechanisms, have been well described (e.g., Bartholow et al. 2011; Bartholow et al. 2003; Casbon et al. 2003; Curtin & Fairchild, 2003; Dougherty et al. 2008; Fillmore et al. 1998; Fillmore et al. 2005; Ridderinkhof et al. 2002; Rose & Duka, 2007, 2008; Vogel-Sprott et al. 2001; Weafer & Fillmore, 2008). Alcohol consumption has been shown to increase the number of errors in tasks that require a high degree of cognitive control, such as a Go/No-Go task (Easdon et al. 2005). In this well-characterized paradigm, participants are required to respond to quickly and frequently presented Go stimuli while suppressing their response to infrequent and task-irrelevant No-Go stimuli. While Go and No-Go stimuli should be similar in visual stimulus processing, No-Go stimuli additionally require the inhibition of a prepotent response.

Studies using event-related brain potentials (ERPs) during Go/No-Go paradigms with non-emotional stimuli typically demonstrate two major ERP components that are enhanced during successful inhibition of a prepotent response (i.e., are larger for No-Go than for Go stimuli) and hence, may represent valuable markers for response inhibition: the N200 and P300 component (Falkenstein et al. 1999). The No-Go N200 is a negative-going component occurring 200–400 ms following stimulus presentation and is maximally at fronto-central scalp locations. The No-Go N200 is believed to index top-down mechanisms needed to inhibit an incorrect tendency to respond on No-Go stimuli (Falkenstein, 2006; Falkenstein et al. 1999; Kaiser et al. 2006; Kopp et al. 1996). However, there is still some debate about the functional specificity of the No-Go N200, since this component has also been associated with the detection of a conflict between initiated and required responses, action monitoring, and effortful attention (Donkers & van Boxtel, 2004; Nieuwenhuis et al. 2003; van Veen & Carter, 2002; Yeung et al. 2004). Although the No-Go N200 might mirror a wide range of cognitive control processes, most authors agree that this component emerges as a result of the employment of cognitive resources involved in inhibitory control.

The No-Go N200 is typically followed by the No-Go P300, which is a positive-going shift with a more central distribution that peaks between 300–600 ms after stimulus presentation (Kopp et al. 1996; Pfefferbaum et al. 1985). In contrast with the N200, the No-Go P300 amplitude has been suggested to be associated with motor inhibition itself (Falkenstein et al. 1999; Smith et al. 2008). In Go/No-Go paradigms, larger No-Go P300 amplitudes seem to reflect the later stage of the inhibitory process that is closely related to the actual inhibition of the motor system in the premotor cortex, rather than the initial reflexive stage of the inhibition (Dimoska et al. 2006; Kok et al. 2004). The N200 and P300 amplitudes during the No-Go conditions are thus considered to represent different sub-processes of response inhibition, and hence, the dysfunction in either or both of these two components may imply the deficiency of cognitive control.

Regarding the effects of alcohol, ERP studies using Go/No-Go paradigms indeed have found that alcohol impairs the ability to inhibit behavior. Furthermore, whereas the effects of acute alcohol on the N200 amplitude are heterogeneous (Curtin and Fairchild, 2003; Easdon et al. 2005; Ridderinkhof et al. 2002) and an earlier study using a non-emotional Go/No-Go paradigm found that the N200 was little affected by a moderate dose of alcohol, alcohol-intoxicated individuals did show reduced P300 amplitudes as compared to sober controls (Easdon et al. 2005). These findings are important because they identify a basic inhibitory mechanism that is impaired by alcohol, which could contribute to the display of impulsive, aggressive, and other socially inappropriate behaviors under the influence of the drug (e.g., Fillmore & Weafer, 2004; Fromme et al. 1997; George & Stoner, 2000; Heinz et al. 2011).

Last years, it became clear that in healthy and sober individuals, cognitive control processes can also be modulated by emotional cues, including emotional faces (e.g., Elliott et al. 2000; Hare et al. 2005; Mathews & MacLeod, 1994; Schulz et al. 2009; Schulz et al. 2007). Compared to neutral stimuli, positive and negative stimuli, such as emotional facial expressions, are recognized faster (e.g., Carjaval et al. 2004), and positive emotions are more readily processed than negative ones as reaction times for happy faces are generally faster than that for negative faces. This positivity bias might be due to the fact that happy faces are less ambiguous (e.g., Carjaval et al. 2004; Eastwood et al. 2003; Leppänen & Hietanen, 2004). Alternatively, it has been suggested that positive stimuli, such as happy facial expressions, facilitate approach tendencies and continued action, thereby making it more difficult to suppress task-inappropriate behaviors (Albert et al. 2010; Schulz et al. 2009). However, these findings appear to conflict with a variety of data showing the existence of a negative bias in emotional processing (i.e., preferential processing or negative cues; Baumeister et al. 2001). Task-irrelevant negative signals have been shown to capture attention more efficiently than task-irrelevant positive signals in behavioral experiments (e.g., Fox et al. 2000). Moreover, ERP studies have revealed that more attention and processing resources are allocated to negative than positive stimuli (Carretié et al. 2001; Smith et al. 2003). Consequently, responses to negative valenced stimuli may be slower as they capture attention and disrupt performance (Eastwood et al. 2003). This seems to be particularly true for responses to angry facial expressions because these cues may provoke extensive and time-consuming cognitive analysis (e.g., Baumeister et al. 2001). Taken together, previous results demonstrate that emotional information, both positive and negative, may be capable of disrupting ongoing cognitive processes by competing for attentional resources with the ongoing task demands (e.g., Chun, 2010; De Houwer & Tibboel, 2010; Pessoa, 2009; Verbruggen & De Houwer, 2007).

More recently, researchers have also employed emotional versions of the Go/No-Go paradigm. This task yields the same measure of cognitive control as a classical Go/No-Go task, but the use of emotional stimuli also permits analysis of performance in response to cues of different emotional valences. This paradigm does not only provide a measure of behavioral inhibition, but also of the emotional modulation of this inhibition (Drevets & Raichle, 1998). Several studies have shown that emotional information is able to modulate cognitive control, both the processes of response inhibition and response execution (Albert et al. 2010; Chiu et al. 2008; Elliott et al. 2004; Elliott et al. 2000; Gopin et al. 2011). Emotional information processing thus seems to play an important role for cognitive control and the execution of appropriate behavior.

In addition to its effect on cognitive control processes, there is compelling evidence that alcohol is also associated with impairments in emotional information processing, resulting in emotional dysregulation and dampened emotional responsiveness. For example, in a study of Franken et al. (2007), ERPs resulting from watching pleasant, unpleasant, and neutral pictures were investigated in a group of participants receiving a beverage containing a moderate dose of alcohol. Results showed that the brain's response to unpleasant emotional pictures was attenuated after ingestion of alcohol, suggesting that alcohol selectively reduced processing of unpleasant stimuli. Moreover, alcohol has also been associated with deficits in facial emotion recognition (e.g., Attwood et al. 2009a; Attwood et al. 2009b; Borrill et al. 1987; Craig et al. 2009; Kano et al. 2003; Tucker & Vuchinich, 1983); however, results are equivocal. In a study of Kano et al. (2003), for example, low doses of alcohol caused a significantly better discrimination of happy faces (i.e., positivity bias), while performance became worse with higher doses. Other studies found that moderate doses of alcohol had significantly higher effect on the perception of anger than with other emotions (Borrill et al. 1987). Furthermore, Orozco et al. (1999) found that alcohol-intoxicated male individuals showed reduced amplitudes on a P300-like ERP component (i.e., with a latency between 400 and 550 ms) to male happy faces. This finding may indicate that deficits in emotional information processing (e.g., facial emotion recognition) in alcohol-intoxicated individuals interfere with the availability and allocation of attentional resources. If applied to measures of cognitive control (such as Go/No-Go paradigms), such facial emotion processing deficits may thus impede response inhibition through reducing the availability of attentional resources, resulting in diminished P300 amplitude for inhibition task demands (Chun, 2010). Arguably, alcohol may lead to a dysregulation of emotional information, which, in turn, makes it more difficult for alcohol-intoxicated individuals to allocate attentional resources for cognitive control processes, resulting in an increased likelihood of an inappropriate behavioral response. However, although there is little dispute that alcohol influences both cognitive control and emotional information processes separately, no direct evidence of the persisting emotional-cognitive interaction exists in individuals under the influence of alcohol.

Therefore, the main focus of the present study is to investigate whether and if so how emotional information influences cognitive control processes and its underlying neural brain mechanisms in alcohol-intoxicated individuals. For this purpose, ERPs generated during an emotional Go/No-Go paradigm with three different types of emotional facial stimuli (angry, happy, and neutral facial expressions) were recorded in individuals under the influence of a moderate dose of alcohol and sober placebo controls. The hypotheses were as follows: First, consistent with the notion that alcohol impairs cognitive control, we expected the number of errors to increase after alcohol consumption. Furthermore, we hypothesized that if alcohol impairs cognitive control, then the N200 and P300 amplitudes should decrease in amplitude after alcohol consumption, and this effect should be greater during No-Go than during Go trials, since only the former requires response inhibition. Second, we hypothesized that emotional information (i.e., emotional faces) would modulate both response execution (Go trials) and response inhibition (No-Go trials) because facial emotion processing should occur preceding to both Go and No-Go trials. Both N200 and P300 amplitudes would be larger for faces with angry or happy emotional facial expressions than those for neutral faces. If, however, emotional information only modulates response execution, then larger N200 and P300 amplitudes for emotional faces would be observed only for Go trials; if emotional information only modulates response inhibition, then larger ERPs for emotional faces would be observed only for No-Go trials. Finally, we hypothesized that the emotional modulation of cognitive control during the emotional Go/No-Go paradigm would characterize the alcohol and placebo groups differentially. Specifically, we expected that the alcohol group would show smaller ERP enhancements in response to emotional facial expressions, which would reflect a reduced availability of attentional resources for inhibition task demands and cognitive control.

## Methods

### Participants

Sixty-four healthy males between the age of 18 and 25 (mean age = 20.51 years, SD = 1.94) were recruited to participate in this study. Participants were recruited from the undergraduate population of the Erasmus University Rotterdam, the Netherlands, and by posting weblogs on social network sites. Screening measures were conducted to determine medical history and took place by telephone. Inclusion criteria were the absence of current medical and psychiatric conditions, and no current use of medication during the past 4 weeks before the experimental session. Participants were not included in the study if they had a self-reported history of alcohol-related problems. The median frequency of drinking days was 9 to 11 days each month, and the median quantity of drinks on each occasion was six glasses. The median age of drinking onset was between 14 and 15 years.

Participants were randomly assigned to an alcohol (*n* = 32) or placebo group (*n* = 32). The alcohol and placebo participants were matched in terms of age. The mean age of the alcohol group was 20.41 years (SD = 1.93), and the mean age of the placebo group was 20.63 year (SD = 1.98). There were no significant group differences on self-reported habitual drinking patterns [total score of the quantity-frequency-variability (QFV) index of drinking patterns] and age of onset of alcohol use (*t*'s(62) < −1.13). Furthermore, no significant pre-existing differences between groups were found in self-reported measures of impulsiveness (BIS-11) and positive and negative affect (PANAS), all *t*'s(62) < 0.91.

### Alcohol dose and beverage administration

Participants in the alcohol group received a moderate dose of alcohol (0.65 g/kg) in a beverage containing one part of vodka (40% of alcohol) and two parts of orange juice, divided equally over two glasses. Participants in the placebo group received a non-alcoholic beverage (0.00 g/kg alcohol), consisting of one part of tonic and two parts of orange juice, which was served in a similar way. To induce an alcohol odor in the placebo group, 4 ml of vodka (1%) was applied on the glasses and floated on top of the beverages. Participants in both groups were told they would receive either a high or a low dose of alcohol. All participants had 2 min to finish each glass through a straw. The two glasses were served 4 min apart (Weafer & Fillmore, 2008).

Subjective effects of drinking were measured 25 min after drinking and immediately following the testing period. Breath samples to measure the blood alcohol concentration (BAC) were collected at five moments during the study using 6510 Alcohol test (Dräger; Lübeck, Germany) breath analyzer equipment: before alcohol administration; 25, 55, and 80 min after beverage administration (immediately preceding and immediately following the testing period); and approximately 2 h after beverage administration. The peak BAC was expected to occur about 60 min after drinking (Fillmore & Vogel-Sprott, 1998; Weafer & Fillmore, 2008).

### Subjective self-report ratings

#### Barratt Impulsiveness Scale-11 (BIS-11)

The Barratt Impulsiveness Scale-11 (BIS-11; Patton et al. 1995) is a self-report measure of impulsiveness and consists of 30 items. For the present study, the summed score of all items was used to determine the degree of impulsiveness; the higher the summed score is, the higher is the level of impulsiveness of the participant. The BIS-11 has good psychometric properties (Patton et al., 1995).

#### The Positive and Negative Affect Scales (PANAS)

The Positive Affect and Negative Affect Scales (PANAS; Watson et al. 1988) consist of 20 items that either measure positive affect (PA; ten items) or negative affect (NA; ten items). Each item refers to a mood state (e.g., proud, scared), and participants rate the extent to which each mood state describes how they feel at the moment of testing on a scale ranging from 1 (not at all or very slightly) to 5 (extremely). The Dutch version of the PANAS has shown excellent psychometric properties (Boon & Peeters, 1999).

#### Habitual drinking patterns (QFV-index)

The QFV-index (Lemmens et al. 1992; Meerkerk et al. 1999) was used to measure habitual drinking patterns. In this questionnaire, three items are employed to determine the drinking quantity (number of glasses), frequency (drinking days), and variability (binge drinking) during the last 6 months. Furthermore, one question was added to determine the age of onset of drinking.

#### Subjective alcohol effects

Throughout the study, participants completed several self-report ratings to assess the subjective effects of the beverage. First, participants indicated on a five-point Likert-scale how many effect they experienced from the beverage (i.e., magnitude of effects; *1 = no effect at all, 2 = a little effect, 3 = moderate effect, 4 = relatively much effect, 5 = strong effect*). Second, a Visual Analogue Scale (VAS) was used to examine their current subjective experience of pleasantness of the effect of the consumed beverage.

### Emotional Go/No-Go task

**Fig. 1:**
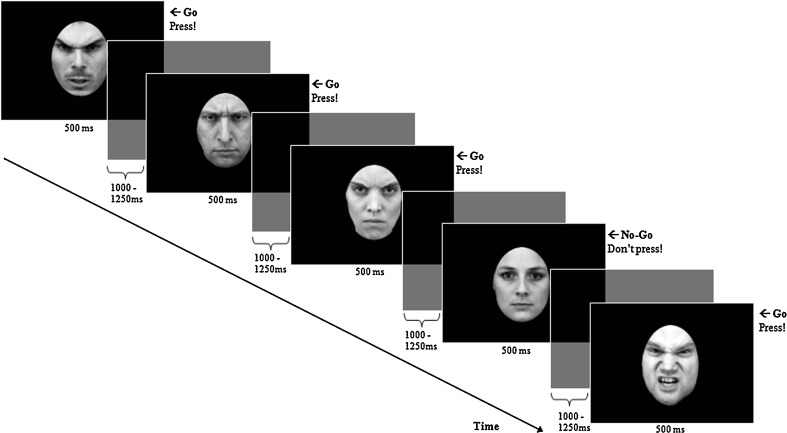
Schematic illustration of five trials of the emotional Go/No-Go task in which angry faces served as Go cues and neutral faces were No-Go cues

Participants completed six blocks of an emotional Go/No-Go task with angry, happy, and neutral facial expressions as targets (Go stimuli) and non-targets (No-Go stimuli), illustrated in Fig. 1. Angry, happy, and neutral pictures of facial expressions from 12 actors (six male and six female actors) were selected from the Karolinska's Directed Emotional Faces (Lundqvist et al. 1998). Based on a recent validation study (Goeleven et al. 2008), actors with the highest recognition hit rate and arousal ratings for angry expressions were chosen. The faces were morphed to mask the hair and cropped in a black oval.

All blocks included only two categories of emotional expressions: one target (Go) and one non-target (No-Go). All combinations of expressions were used as both targets and non-targets. Each block contained 84 stimuli, of which 63 (75%) were Go cues and 21 (25%) were No-Go cues. This resulted in a total of 504 trials, of which 378 were Go cues and 126 were No-Go cues. Consequently, there were 126 Go cues and 42 No-Go cues for each emotional condition. Order of the blocks was randomized across subjects, and the order of the trials was pseudorandomized within each block to control for order of presentation (i.e., avoiding the consecutive presentation of two No-Go trials within each block).

Participants were first given the opportunity to practice in a block of 16 “neutral is Go” trials. Once it was determined that they could perform the task, the experimental trials started. Before each run, participants were given instructions to respond to a particular facial expression (Go trials) by pressing a button with their index finger of their dominant hand and to withhold responses for any other expression (No-Go trials). They were required to respond as fast as possible without making mistakes. Each facial stimulus was presented for 500 ms in the middle of a black screen. The intertrial interval was varied between 1,000 and 1,250 ms.

For the current study, the number of commission errors on No-Go trials (i.e., false alarms) and the number of omission errors on Go trials across the whole task and on each of the three emotional facial conditions served as primary measures of behavioral inhibition and execution, respectively, and the emotional modulation of these processes. The mean reaction time (RT) on correct Go trials for the emotional facial conditions was used as a measure of emotional bias (e.g., Elliott et al. 2004; Elliott et al. 2000; Hare et al. 2005).

### Procedure

Interested volunteers responded to study advertisements and weblogs on social network sites by contacting us by email or telephone. A screening by telephone was conducted during which students received information about the experimental procedure and in order to determine eligibility for participation. Eligible volunteers then made appointments to come to the Erasmus Behavioral Lab (Erasmus University Rotterdam) for one experimental session of approximately 2 h. All participants were instructed to abstain from food and cigarette use 2 h before testing and to abstain from caffeine on the testing day, as well as to refrain from consuming alcoholic beverages or any psychoactive drugs or medications for 24 h before the experimental session. At arrival at the laboratory (all between noon and 6 p.m.), participants signed informed consent, they were weighed, and initial breath alcohol level was assessed to ensure that they were sober before onset of the experiment. None of the participants was positive on this test. Hereafter, participants completed the self-report questionnaires. Subsequently, the alcohol or placebo beverage was administered, and subjects were seated on a comfortable chair in a light and sound-attenuated room. After the EEG electrodes were attached (which took 25 min), BAC was monitored for the second time, and participants performed the emotional Go/No-Go task, lasting about 20 min. Then, they completed a brief subjective measure of the magnitude and the pleasantness of the alcohol effects, and BAC level was monitored for the third time. Approximately 50 min after alcohol administration, a task was administered to measure risky decision making and feedback processing (results reported elsewhere). Hereafter, participants were disconnected from the EEG. They were given a final subjective measure of alcohol effects, and BAC level was measured for the fourth time. Participants were then instructed to remain in the university building and had to go to the refectory to eat and drink something. After 45 min (i.e., 2 h after beverage administration), participants had to return to the lab to monitor their final BAC, and participants were allowed to leave once their BAC fell to 20 mg/100 ml or below. All participants received a small financial compensation or course credits for their participation, and they were instructed not to drive a vehicle after the experiment. The study was conducted in accordance with the Declaration of Helsinki and approved by the ethics committee of the Institute of Psychology of the Erasmus University Rotterdam.

### Electrophysiological recording and analysis

The EEG was recorded with BioSemi Active-Two using 34 scalp sites (10–10 system, and two additional electrodes at FCz and CPz) with Ag/AgCl active electrodes mounted in an elastic cap. Furthermore, six additional electrodes were attached. Two electrodes were attached to the left and right mastoids as reference electrodes. To record ocular movement and to be able to correct for ocular artifact, two electrodes were placed next to each eye for horizontal electrooculogram (HEOG), and two electrodes were placed above and below the left eye for vertical electrooculogram (VEOG). Online signals were recorded with a low-pass filter of 134 Hz. All signals were digitized with a sample rate of 512 Hz and 24-bit A/D conversion. Data were offline referenced to mathematically linked mastoids.

EEG data were filtered offline using a conventional wide band filter of 0.10 to 30 Hz (phase shift-free Butterworth filters; 24-dB/octave slope). Stimulus-locked epochs were computed −200 to 800 ms from stimulus onset. After ocular correction (Gratton et al. 1983), epochs, including out of range voltages (± 75 μV), were rejected as artifacts and were excluded from further processing. Individual ERP averages were derived for correct trials of each stimulus type (Go, No-Go) and emotional facial expression (angry, happy, neutral) and were baseline corrected using the 200-ms pre-stimulus interval. Segments with incorrect responses (miss for Go trials or false alarm for No-Go trials) were excluded from analyses. If 50% or more of the epochs of a participant contained artifacts, this participant was excluded from ERP analyses. As a result, six participants were rejected from ERP analyses, four in the placebo group, and two in the alcohol group. The mean number of analyzable Go and No-Go epochs for angry facial expressions was 94.0 and 25.8, for happy facial expressions 102.8 and 25.7, and for neutral facial expressions 93.2 and 27.7, respectively.

Derived from inspecting grand average and individual subject data, the N200 component was identified by using an area measure capturing the average activity between 300 and 400 ms following stimulus onset and preceding the P300, which is typical in the context of the present paradigm. A time-window measuring the average activity between 400 and 600 ms was used to define the P300 component. Statistical analyses for both components were done on fronto-central electrodes, including Fz, FCz, FC1, FC2, and Cz.

### Statistical analyses

#### Demographic differences

Demographic differences between groups with respect to age and scores on the subjective self-report ratings were assessed with independent samples *t*-tests. Independent samples *t*-tests were also conducted to assess differences in BAC levels before and after administration of the emotional Go/No-Go task.

#### Behavioral analysis

Commission and omission errors (i.e., button presses in No-Go trials [false alarms] and no responses in Go trials, respectively) and reaction times (RTs) to correct Go trials (i.e., hits) on the emotional Go/No-Go task were calculated for all face trials (regardless of emotional expression) and separately for trials with angry, happy, and neutral emotional facial expressions. As additional measures of accuracy, we also calculated the signal detection measures *d'* (sensitivity or discriminability) and criterion *C* (response bias; Macmillan and Creelman, 2005) for all emotional Go/No-Go combinations in each block. Sensitivity (*d')* was calculated as the difference between the z-score transformation of the hit (proportion of all valid Go stimuli that were responded to) and false alarm rate (proportion of all valid No-Go stimuli that were incorrectly responded to, [*d'* = *z*(*hits*) – *z*(*false alarms*)]). This measure represents the sensitivity to different stimulus conditions that is independent from respondent biases (i.e., the ability to discriminate target from non-target stimuli). Response bias *C* was calculated using the formula *–0.5*[*z(hits) + z(false alarms)*], and represents participant's tendency to respond to task items, regardless of whether they are correct. Hence, response bias reflects the minimum level of internal certainty needed to decide that a particular stimulus is present. A neutral criterion or the absence of response bias is present if *C* = 0. A liberal response bias (i.e., as participants are more willing to respond yes) results in *C* < 0, whereas a conservative response bias (i.e., less willing to respond yes) results in *C* > 0. In our paradigm, this *C* statistic will also provide a measure of response bias towards angry, happy, or neutral facial expressions, with lower values indicating greater bias. As there were instances in which participants had perfect performance (i.e., 1 or 0 for hits or false alarms), which can result in statistically infinite *d'*, we transformed the hit and false alarm rates for each participant using a log-linear rule before calculating *d*' scores (Hautus, 1995). We adjusted the scores to avoid infinite *d*' by adding 0.5 to all the cells and dividing the resulting scores by the number of trials (*n* + 1) related to the proportion (Macmillan & Creelman, 2005). Thus, hit and false alarm rates were calculated using the formula [*hits + 0.5/(hits + misses + 1)*] and [*false alarms + 0.5/(false alarms + correct rejections + 1)*], respectively.

To assess the effects of alcohol, emotion, and trial type on response inhibition, a 2 × 2 × 3 repeated measures ANOVA was carried out on error rates with *Group* as between-subject factor (alcohol, placebo) and *Trial Type* (Go, No-Go) and *Emotion* (angry, happy, neutral) as within-subjects factors. With respect to RTs to correct Go trials, a 2 × 3 repeated measures ANOVA was performed with *Group* (alcohol, placebo) as between-subject factor and *Emotion* (angry, happy, neutral) as within-subjects factor. The signal detection measures were analyzed by performing a 2 × 6 repeated measures ANOVA with *Group* as between-subject factor and *Block* (the six emotional Go/No-Go combinations in each block) as within-subject factor.

#### ERP analyses

To assess the effects of alcohol, response inhibition, and emotion on brain activity, 2 × 2 × 3 × 5 repeated measures ANOVAs were performed for mean N200 and P300 amplitudes, respectively, with *Group* (alcohol, placebo) as between-subject factor and *Trial Type* (Go, No-Go), *Emotion* (angry, happy, neutral), and *Electrode Site* (5 fronto-central electrodes: Fz, FCz, FC1, FC2, and Cz) as within-subject factors. Greenhouse–Geisser corrections were adopted where appropriate. All significant ANOVA effects were further analyzed using Bonferroni-corrected post-hoc *t*-tests. Post-hoc tests for interactions were performed only for interactions including the between-subject factor Group.

#### Correlational analyses

Bivariate correlation analyses using Pearson's correlation coefficient were computed to examine associations between behavioral measures and electrophysiological indices of cognitive in the alcohol and placebo groups separately. For all analyses, two-tailed tests were used, and a 0.05 level of significance was employed.

## Results

### Blood alcohol concentration and subjective alcohol effects

No detectable BACs were observed in the placebo group (i.e., 0.00‰). In the alcohol group, the mean BAC level 25 min after initiation of drinking alcohol (BAC2) and just before the start of the emotional Go/No-Go task was 0.73‰ (SD = 0.18; range = 0.39–1.12‰). The mean BAC level a further 20 min later (BAC 3) after completion of the task was 0.77‰ (SD = 0.13; range = 0.52–0.97‰).

With respect to the subjective experience of the effects of the drinks, there was a significant difference (*t*(62) = 7.57, *p* < .001) in mean magnitude of the alcohol effects between the alcohol group (*M* = 3.34, SD = 0.94) and the placebo group (*M* = 1.72, SD = 0.77) before administration of the emotional Go/No-Go, and this difference remained significant after completing the task (*t*(62) = 6.00, *p* < .001). Regarding the pleasantness ratings of the effect of the drinks, the alcohol group (*M* = 68.25, SD = 16.96) rated the drink as more pleasant (*t*(62) = 2.99, *p* < .01) than the placebo group (*M* = 55.06, SD = 18.30).

### Behavioral results

#### Error rates

Table 1 presents the commission and omission error rates and RTs for Go trials on the emotional Go/No-Go task for the alcohol and placebo groups separately. A robust main effect of Trial Type (*F*(1, 62) = 66.25, *p* < .001) revealed that overall, participants were less accurate on No-Go trials (i.e., commission errors, *M* = 25.3%) than on Go trials (i.e., omission errors, *M* = 7.8%). A significant main effect of Emotion emerged (*F*(2, 124) = 8.14, *p* = .001) due to higher overall error rates on angry emotional face trials (*M* = 19.3%) relative to both happy (*M* = 14.5%) and neutral face trials (*M* = 15.8%; angry vs. happy, *p* < .001; angry vs. neutral, *p* = .05; happy vs. neutral, *p* = .70). The main effect of Emotion was qualified by a significant Emotion × Trial Type interaction-effect (*F*(2, 124) = 8.69, *p* = .001). Within No-Go trials, response inhibition to angry faces was less accurate (i.e., participants made more commission errors) than neutral faces (angry vs. neutral, *p* < .05). There was no response difference between angry and happy faces (angry vs. happy, *p* = .48; happy vs. neutral, *p* = .21). Among Go trials, responses to angry faces were less accurate than happy faces, but not to neutral faces (angry vs. happy, *p* < .01; angry vs. neutral, *p* = .55; happy vs. neutral, *p* = .001).

**Table 1:** Behavioral results of cognitive control during the emotional Go/No-Go task for the alcohol and placebo groups

Group	Variables	Emotional Go/No-Go							
		Angry faces		Happy faces		Neutral faces		All faces	
		Mean	SD	Mean	SD	Mean	SD	Mean	SD
Alcohol	CE (%)	34.60	17.19	31.55	19.36	27.90	18.15	31.35	16.36
	OE (%)	12.25	13.34	4.17	5.56	6.62	9.59	7.68	6.29
	Go RT (ms)	395.1	56.0	361.1	38.8	375.8	47.0	377.3	42.2
Placebo	CE (%)	20.24	12.76	19.57	12.05	17.86	13.36	19.22	11.26
	OE (%)	10.27	12.07	2.80	4.33	10.69	13.19	7.92	7.81
	Go RT (ms)	410.4	55.9	384.2	64.6	410.5	61.7	401.7	58.2

*CE* percentage of commission errors (i.e., the number of times the participant responded to a No-Go trial divided by the total number of No-Go trials), *OE* percentage of omission errors (i.e., the number of times the participant did not respond to a Go trial divided by the total number of Go trials), *RT* reaction time correct Go trials, *ms* milliseconds

With respect to the effect of acute alcohol intoxication, a robust main effect of Group (*F*(1, 62) = 11.32, *p* = .001) showed that overall task performance was less accurate in alcohol-intoxicated participants as compared to sober controls (*M* error rates 19.5% versus 13.6%, respectively). Furthermore, a significant Group × Trial Type interaction-effect was found (*F*(1, 62) = 8.29, *p* < .01). Parsing of this interaction revealed that participants in the alcohol group were less accurate only among No-Go trials (i.e., commission errors) as compared to the placebo group (*M* = 31.4% versus *M* = 19.2%, *p* = .001, respectively), and this group difference was absent in Go trials (i.e., omission errors; *M* = 7.7% and *M* = 7.9%, *p* = .90, respectively). The interaction-effects of Group × Emotion or Condition × Emotion × Trial Type did not reach statistical significance (*p*'s > .11).

#### Reaction time

Reaction time data showed a significant main effect for Emotion (*F*(2, 124) = 24.55, *p* < .001), indicating that participants were quicker to respond to happy face trials (*M* = 372.7 ms) relative to angry and neutral face trials (402.7 and 393.2 ms, respectively; angry vs. happy, *p* < .001; angry vs. neutral, *p* = .17; happy vs. neutral, *p* < .001). Moreover, a marginally significant main effect of Group was found (*F*(1, 62) = 3.67, *p* = .06). Post-hoc analysis revealed that alcohol-intoxicated participants tended to react faster on Go trials as compared to sober controls (377.3 vs. 401.7 ms, respectively). No significant Group × Emotion interaction-effect was found on reaction times (*F*(2, 124) = 2.46, *p* = .09).

#### Signal detection measures

**Fig. 2:**
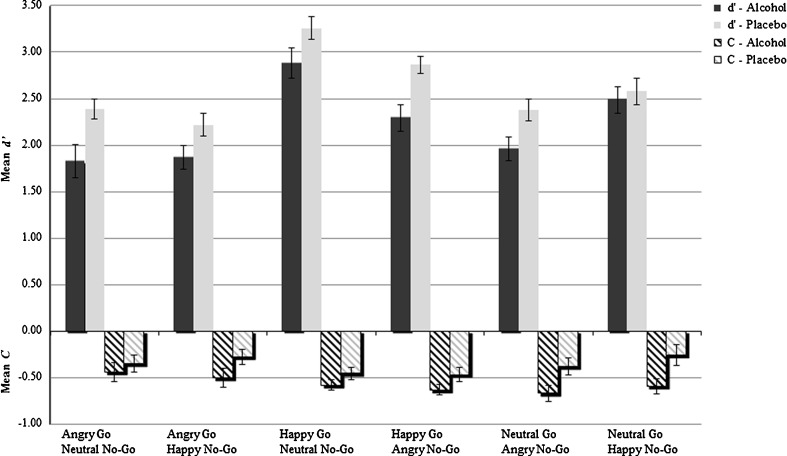
Graphical display of the mean value of sensitivity (*d*') and response bias (*C*) for the alcohol and placebo groups for the six different emotional Go/No-Go combinations presented in each block of the Go/No-Go task. *Error bars* represent SE

Results are graphically displayed in Fig. 2. With respect to sensitivity *d*', a significant main effect of Block could be observed (*F*(5, 310) = 26.87, *p* < .001). Post-hoc analysis revealed that sensitivity (i.e., discrimination ability) was highest in the happy Go neutral No-Go block as compared to all other Go/No-Go combination blocks (*p* < .001), whereas *d*' was most reduced in the blocks were angry faces served as Go trials, as well as in the neutral Go angry No-Go block. Furthermore, a significant main effect of group could be observed (*F*(1, 62) = 9.20, *p* < .01), indicating that overall, alcohol-intoxicated participants exhibited a reduced sensitivity (*d*') to all facial expressions as compared to sober controls (2.22 vs. 2.61, respectively). The Group × Block interaction-effect did not reach statistical significance. Differences in response bias *C* on emotional facial expressions in the six blocks just missed significance (*F*(5, 310) = 2.28, *p* = .06). However, we did observe a significant main effect of Group (*F*(1, 62) = 5.86, *p* < .05), indicating that alcohol-intoxicated participants exhibited a greater response bias (as indexed by lower criterion *C*) to all facial expressions as compared to sober controls (−0.57 vs. −0.37, respectively). Again, no significant Group × Block interaction-effect was found.

### Electrophysiological results

#### N200 amplitude

**Fig. 3:**
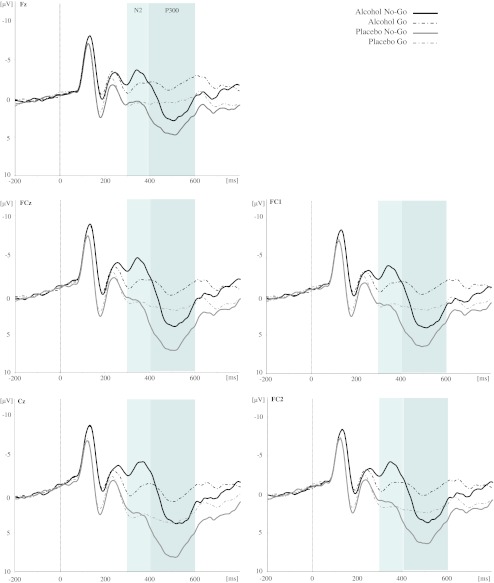
Stimulus-locked grand average waveforms from the five electrode sites (Fz, FCz, Cz, FC1, and FC2, respectively) evoked by the total number of Go and No-Go trials (averaged across emotions) in the emotional Go/No-Go task as a function of Group (alcohol versus placebo)

**Fig. 4:**
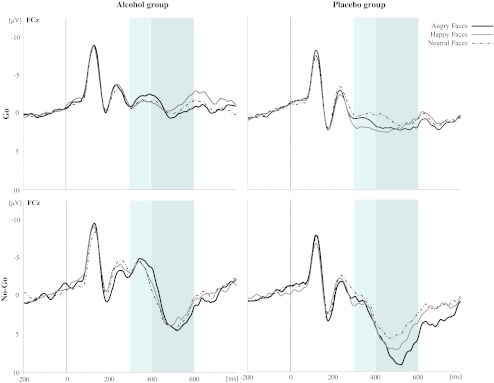
Stimulus-locked grand average waveforms from electrode FCz (*left*) evoked by Go trials (*upper panel*) and No-Go trials (*bottom panel*) in the emotional Go/No-Go task as a function of Group (*left panel*: alcohol group, *right panel*: placebo group) and Emotion (angry, happy, and neutral faces)

Mean N200 amplitudes for both groups for each trial type (Go, No-Go) and emotional facial expression (angry, happy, neutral) are presented in Table 2. A selection of grand averages for each group as a function of trial type and emotional facial expression is shown in Figs. 3 and 4, respectively. As expected, a main effect was found for Trial Type (*F*(1, 56) = 7.39, *p* < .01; Fig. 3) on the N200 amplitudes at the fronto-central cluster of electrodes showing that N200 amplitudes were larger for No-Go trials than for Go trials (−0.89 μV vs. 0.06 μV, respectively). We also observed a significant main effect of Electrode Site (*F*(4, 224) = 19.43, *p* < .001), indicating that N200 was largest in Fz and FCz (−0.97 mV and −0.77 μV, respectively) followed by all other electrodes (all *p*'s < .05). Furthermore, a significant main effect was found for Emotion (*F*(2, 112) = 3.78, *p* < .05), indicating that overall, happy faces elicited the least pronounces N200 amplitudes (0.05 μV) relative to neutral and angry facial expressions (−0.63 versus −0.67 μV, respectively; happy vs. neutral, *p* < .05; happy vs. angry, *p* = .08; angry vs. neutral, *p* = 1).

**Table 2:** Mean N2 and P300 peak amplitudes on the emotional Go/No-Go task for the alcohol and placebo groups

Trial type	Emotion	Alcohol group (*n* = 30)				Placebo group (*n* = 28)			
		N200		P300		N200		P300	
		Mean	SD	Mean	SD	Mean	SD	Mean	SD
“Go”	Angry	−1.69	4.26	−0.58	4.03	1.12	3.40	2.22	3.28
	Happy	−0.90	4.20	−1.11	4.21	2.27	2.90	1.96	2.47
	Neutral	−1.10	4.48	−0.84	4.03	0.66	4.13	1.14	3.33
	Total Go	−1.23	4.07	−0.85	3.80	1.35	3.10	1.77	2.66
“No-Go”	Angry	−3.58	5.26	2.00	6.51	1.48	4.18	6.60	4.62
	Happy	−3.10	5.58	1.91	6.03	1.94	5.94	5.47	5.28
	Neutral	−3.05	4.97	2.42	5.39	0.97	5.03	4.12	5.47
	Total No-Go	−3.24	4.74	2.11	5.42	1.47	4.24	5.40	4.57

N200 and P300 are mean amplitudes in microvolt averaged across five recorded fronto-central scalp sites (Fz, FCz, FC1, FC2, and Cz)

With respect to the effects of acute alcohol intoxication, a robust main effect was found for Group (*F*(1, 56) = 12.82, *p* = .001). Overall, alcohol-intoxicated individuals demonstrated larger N200 amplitudes as compared to sober controls (−2.24 vs. 1.41 μV, respectively). This effect was qualified by a significant Group × Trial Type interaction-effect (*F*(1, 56) = 9.32, *p* < .01). Post-hoc analysis indicated that in the alcohol group, N200 amplitude was significantly larger for No-Go trials as compared to Go trials (*p* < .001), whereas this difference in the placebo group was absent (*p* = .82). Finally, a significant Group × Emotion × Trial Type × Site interaction-effect was observed (*F*(8, 448) = 2.60, *p* < .05). Parsing of this interaction revealed that the difference between Go and No-Go trials in the alcohol group was significant at all electrode sites except for Fz (*p* > .09). Post-hoc analysis further revealed that the N200 amplitude differences between groups were significant for all emotional Go and No-Go trials, except for the neutral Go condition (for each electrode site; all *p*'s > .08). Furthermore, pairwise comparisons demonstrated that in the alcohol group, for all electrode sites, Go and No-Go N200 amplitudes did not differ between emotional facial expressions (all *p*'s > .10), whereas emotion in the placebo group only modulated the N200 in response to Go trials (i.e., neutral and angry facial expression elicited larger Go-N200 amplitudes relative to happy faces at FC1 and FC2; at sites Fz, Cz, and FCz, the difference between angry and happy faces did not reach statistical significance). In contrast, inhibitory control, as indexed by the early N200 in response to No-Go trials, was not modulated by emotion.

#### P300 amplitude

Mean P300 amplitudes for both groups for each trial type (Go, No-Go) and emotional facial expression (angry, happy, neutral) are presented in Table 2. A selection of grand averages is shown in Figs. 3 and 4. As expected, a robust main effect was found for Trial Type (*F*(1, 56) = 53.60, *p* < .001) on P300 amplitudes at the fronto-central cluster of electrodes, showing that P300 amplitudes were larger for No-Go trials than for Go trials (3.75 vs. 0.46 μV, respectively). We also observed a significant main effect of Electrode Site (*F*(4, 224) = 50.92, *p* < .001), indicating that P300 amplitude was significantly larger at Cz (3.09 μV) than at all other electrodes (all *p*'s < .01). Furthermore, a significant main effect of Emotion emerged (*F*(2, 112) = 4.11, *p* < .05) due to enhanced overall P300 amplitudes elicited by angry facial expressions (2.56 μV) relative to neutral facial expression (1.71 μV; angry vs. neutral, *p* < .05; angry vs. happy, *p* = .26; happy vs. neutral, *p* = .62).

Regarding the effects of acute alcohol intoxication, a main effect was found for Group (*F*(1, 56) = 8.34, *p* < .01). Overall, alcohol-intoxicated participants demonstrated significantly smaller P300 amplitudes as compared to placebo controls (0.63 vs. 3.58 μV, respectively). This effect was qualified by a significant Group × Trial Type × Site interaction-effect (*F*(4, 224) = 3.68, *p* < .05). Parsing of this interaction revealed that among Go trials, alcohol and placebo participants differed significantly at all electrode sites, whereas among No-Go trials, this effect was absent at electrode site Fz (*p* = .08). Most interestingly, however, the main effect of Group was qualified by a significant Group × Emotion interaction-effect (*F*(2, 112) = 6.10, *p* < .01). Post-hoc analysis revealed that the P300 amplitudes of the alcohol and placebo participants differed in response to both angry and happy facial expressions (*p* = .001 and *p* < .01, respectively) and that there was no significant P300 amplitude between-group difference in response to neutral facial expressions (*p* = .08). Furthermore, pairwise comparisons demonstrated that in the placebo group, P300 amplitudes were significantly larger for angry and happy emotional facial expressions as compared to neutral expressions (angry vs. neutral, *p* = .001; happy vs. neutral, *p* < .05; angry vs. happy, *p* = .30), whereas P300 amplitude in the alcohol group did not differ between the different emotional facial expressions (all *p*'s > .91). No other significant interaction-effects including Group were found for P300 amplitude (all *p*'s > .22).

### Negative shift after alcohol intoxication

As can be seen in Fig. 3, ERPs from the alcohol-intoxicated participants showed a broad and long-lasting post-stimulus negative deflection (i.e., a negative shift) relative to all emotional Go and No-Go amplitudes as compared to the placebo group, which may suggest that the group differences of No-Go N200 and P300 ERPs might be due to a general cognitive decline after alcohol consumption. This was evidenced by examining the brain activity as the mean value in the 0–800-ms time window after onset of the stimulus. Particularly, when averaging all Go and No-Go trials across emotions for each electrode site over the entire recording time-window, alcohol-intoxicated participants showed significant overall reductions in brain activity (−1.41 vs. 0.86 μV for placebo controls; *t*(56) = −3.61, *p* = .001). Furthermore, regarding the P300, the Go stimuli also elicited P300 amplitudes (reflecting response execution) reliably for all participants and showed smaller amplitudes for alcohol-intoxicated participants.

Therefore, to underline the group difference of the Go/No-Go effect, we additionally calculated the difference wave of N200 (=Nd200) and P300 (=Pd300) by subtracting the Go trials from the No-Go trials for each emotional facial condition. This difference wave might further specifically reflect inhibitory functioning. Repeated measures ANOVAs of Nd200 and Pd300 amplitudes were calculated for between-group comparisons with the factors of *Group* (alcohol vs. placebo), *Emotion* (angry, happy, neutral), and *Electrode Site* (Fz, FCz, Cz, FC1, and FC2). With respect to Nd200, a significant main effect of Group (*F*(1,56) = 9.32, *p* < .01) could be observed, indicating larger Nd200 amplitudes for the alcohol group than the placebo controls (−2.02 vs. 0.12 μV, respectively). Hence, the negative shift did not account for the quite unexpected result of greater N200 in the alcohol group. In contrast, no difference in the amplitude of the Pd300 were found (*F*(1, 56) = 0.56, *p* = .50). Emotion did not reveal significant modulation effects specifically on inhibitory functioning.

### Correlational results

Correlations between behavioral measures and electrophysiological indices of cognitive control are presented in Table 3. For both groups, shorter RTs were associated with more commission errors (CE) and less omission errors (OE). Interestingly, associations between ERPs and the behavioral measures of cognitive control varied by group. In the placebo group, response execution (i.e., percentage of OE) was significantly associated with Go P300 amplitudes and marginally significant with No-Go P300 (*p* = .07). Specifically, the higher the error rates are, the smaller are the P300 amplitudes; however, this relationship was absent in the alcohol group (test of differences in correlations: *Z* = 1.64, *p* one-tailed = .05). Furthermore, No-Go N200 amplitudes in the placebo group were positively associated with response execution (i.e., percentage of OE; *Z* = -1.86, *p* = .03) and inversely correlated with RTs on correct Go trials, whereas these relationships were absent in the alcohol group. However, the latter comparison of correlations did not reveal significant differences between groups (*Z* = 1.41, *p* = .08).

**Table 3:** Correlations between behavioral and electrophysiological measures of cognitive control in the alcohol and placebo groups

	Alcohol group (*n* = 30)							Placebo group (*n* = 28)						
Variable	CE total	OE total	Go RT	N200 Go	N200 No-Go	P300 Go	P300 No-Go	CE total	OE total	Go RT	N200 Go	N200 No-Go	P300 Go	P300 No-Go
CE total		−0.19	−0.39^a^	0.03	−0.05	−0.27	−0.31		−0.33	−0.63^b^	0.28	0.43^a^	0.13	0.37
OE total	−0.19		0.47^b^	−0.24	0.04	−0.01	−0.05	−0.33		0.67^b^	−0.37	−0.19	−0.43^a^	−0.35
Go RT	−0.39^a^	0.47^b^		−0.32	−0.16	−0.03	−0.15	−0.67^b^	0.63^b^		−0.25	−0.50^b^	−0.05	−0.36

For the N200 and P300, mean amplitudes averaged across five recorded fronto-central scalp sites (Fz, FCz, FC1, FC2, and Cz) were used for total Go and No-Go trials (averaged across the three emotional facial conditions)

*CE* commission errors, *OE* omission errors, *Go RT* reaction time on correct Go trials

^a^Correlation is significant at the 0.05 level

^b^Correlation is significant at the 0.01 level

## Discussion

Alcohol has been widely associated with impairments in cognitive control and emotional dysregulation, with consequent effects on behavior. Determining how cognition and emotion interact is pivotal to an understanding of the socially maladaptive behaviors frequently seen in alcohol-intoxicated individuals. This is the first study that we know that directly investigated whether and if so how acute alcohol intake affects the emotional modulation of cognitive control and its underlying neural brain mechanisms by using an emotional Go/No-Go task. Overall, participants across groups made more errors and showed significantly enlarged N200 and P300 amplitudes during No-Go trials as compared to Go trials, suggesting that the task was valid: It did establish a prepotent response, and this response was difficult to inhibit. More importantly, our study provides clear evidence (a) that alcohol impaired the behavioral performance, as well as the early (N200) and later (P300) electrophysiological indices of cognitive control (hypothesis 1), (b) that emotional information has a modulatory effect on both behavior, as well as on the electrophysiological indices of cognitive control (hypothesis 2), and (c) that alcohol affected these emotion modulation effects (hypothesis 3).

On the behavioral level, we found significant effects of alcohol and emotion on cognitive control, but no interaction effect could be observed. Results revealed higher overall error rates in response to angry emotional faces, whereas happy faces elicited the fastest responses to correct Go trials. These results might be explained by dimensional models of emotion that postulate that negative emotions have been directly associated with avoidance behaviors (i.e., withdrawal motivation), whereas positive emotions have been directly associated with approach motivational tendencies and continued action (e.g., Ashby et al. 1999; Cacioppo & Gardner, 1999; Rowe et al. 2007; Seidel et al. 2010). In this sense, it is not surprising that participants responded faster to correct Go trials following happy faces. However, growing evidence suggests that anger (unlike fear) is actually an approach-related negative affect that is also associated with approach motivational tendencies (Harmon-Jones et al. 2009; for review, see Carver & Harmon-Jones, 2009), and this may explain our finding of increased error rates in response to angry faces. Given the social relevance of angry faces and the potential cost associated with failing to notice an angry or threatening face, it is plausible that angry faces might capture attention more effectively than positive faces. Indeed, results of previous studies suggest that negative facial expressions are capable of capturing attention and interfering with ongoing task performance, even when emotional expression is irrelevant to the task demands (e.g., Eastwood et al. 2001, 2003; Fox et al. 2000; Vuilleumier et al. 2001). The present findings also support the conclusion that negative faces are more effective at involuntarily attracting or capturing attention than positive faces, thereby making it more difficult to appropriately respond to the task demands.

More importantly, however, acute alcohol intake impaired the ability to suppress a prepotent response, irrespective of emotional content, whereas alcohol had no significant effect on the ability to respond on Go trials, which is consistent with previous findings (e.g., Easdon et al. 2005). In addition, the alcohol group tended to react faster than controls, implying a speed-accuracy trade off, with alcohol-intoxicated individuals showing more impulsive responding. Together, these findings show that acute alcohol intake results in impaired behavioral inhibitory control.

With respect to the electrophysiological findings, we found differences in early versus later cognitive control processes, since N200 and P300 amplitudes did not show a similar sensitivity to the effects of emotional information and alcohol. Emotion only modulated the N200 in response to Go trials in placebo controls (i.e., response execution, where neutral and angry facial expressions elicited larger Go-N200 amplitudes as compared to happy faces), whereas this emotional modulation effect was absent in the alcohol group. Inhibitory control, as indexed by the early N200 in response to No-Go trials, was not modulated by emotional valence, suggesting that angry, happy, and neutral emotional faces established similar prepotent tendencies.

Interestingly, No-Go N200 amplitudes were found to differ significantly between groups, with alcohol-intoxicated individuals displaying an enhanced (i.e., more negative) No-Go N200 amplitude as compared to sober controls. These results are in contrast with the findings of Easdon et al. (2005) and Ridderinkhof et al. (2002), where the N200 amplitude was not modulated by alcohol, and also contradict the results of Curtin and Fairchild (2003), who did observe a reduced (as compared to enhanced) N200-like wave when intoxicated. However, our results are in line with some previous studies examining individuals with ADHD and depression (Prox et al. 2007; Smith et al. 2004; Zhang et al. 2007) and heroin addicts (Yang et al. 2009). Nevertheless, the enhanced No-Go N200 in alcohol-intoxicated individuals seems at odds with and cannot be easily explained by the common hypothesis that the No-Go N200 reflects inhibitory processes. According to this inhibition hypothesis (Falkenstein et al. 1999), enhanced No-Go N200 amplitudes are related to more efficient inhibition (i.e., less commission errors). In our study, however, a significantly larger No-Go N200 effect was observed in the alcohol group, though commission error rates in this group were found to be higher than in placebo controls. Furthermore, correlational results indicated that in our study, No-Go N200 amplitudes in the alcohol group were unrelated to behavioral inhibition (i.e., the number of commission errors). Hence, an important question to be discussed is the nature of the neural brain mechanisms underlying No-Go N200.

Considerable evidence suggests that early ERP components reflect attentional processes triggered by task demands (Fabiani et al. 2007). In this case, when comparing the current findings to the previously published results, the nature of the employed task (i.e., differences in task demands) and the added emotional component in our paradigm (i.e., increased complexity of the face stimuli) may also explain the fact that our N200 results are not equivalent to the previous findings (e.g., Curtin & Fairchild 2003; Easdon et al. 2005; Ridderinkhof et al. 2002). In a study of Falkenstein et al. (1999), it was shown that the N200 amplitude is enlarged for successful inhibiters (i.e., less commission errors during No-Go trials) as compared to poor inhibiters, which was interpreted as being due to increased effort by successful inhibiters. The larger N200 amplitudes after alcohol consumption in our study may thus be due to increased effort to discriminate between signal and noise trials (i.e., Go and No-Go trials), which was also evidenced by the reduced signal detection parameters in the alcohol group (i.e., reduced sensitivity and a greater response bias to all facial expressions). Hence, one theoretical implication arising from our study is that the mechanism for prepotent response inhibition in alcohol-intoxicated individuals during an emotional Go/No-Go task seems to be intact, but needs to be triggered more strongly than in controls, and that more effort is engaged or required in order to inhibit a prepotent response. Nonetheless, they still fail to achieve the same level of behavioral performance.

Another explanation, however, may be that the N200 reflects conflict monitoring processing rather that inhibitory control, since our results appear more consistent with the conflict-detection theory of N200 (Donkers & van Boxtel, 2004; Nieuwenhuis et al. 2003). According to this theory, the N200 in Go/No-Go tasks reflects conflict arising from competition between the execution and inhibition of a single response (Nieuwenhuis et al. 2003). In our study, conflict occurred when a response must be suppressed (No-Go) in a situation in which there is a prepotent tendency to make a response (Go). This conflict is present whether or not participants can inhibit their motor response. Accordingly, alcohol may have affected the N200 due to the effect that more conflict was present. Specifically, in alcohol-intoxicated individuals, more erroneous activation of the Go response on No-Go trials yields more response conflict on No-Go trials—which is supported by the higher rate of commission errors—enlarging the No-Go N200.

Taken together, it is clear from the results of our study that the N200 process in alcoholic-intoxicated individuals differs from placebo controls and that alcohol is associated with abnormal early cognitive control processes. We think that it is safe to conclude that the larger No-Go N200 in alcohol-intoxicated individuals reflects increased levels of effortful attention required to effortfully regulate a response, either to inhibit a prepotent response or to monitor conflict. This issue requires further investigation.

In contrast to the No-Go N200, there is considerable consensus in the literature that the No-Go P300 amplitude is associated with the inhibitory process itself (e.g., Smith et al. 2008). In our study, the P300 component was found to be modulated by alcohol, as well as by emotional information. Particularly, enhanced overall P300 amplitudes were found following angry facial expressions, suggesting that more effortful attention or further processing may have been required to perform the task (i.e., for response inhibition as well as execution) when confronted with an angry face. However, our results also demonstrate an emotion modulation effect on Go P300 amplitudes, suggesting that both response inhibition and execution were influenced by emotional facial expressions.

With respect to the acute effects of alcohol on P300 amplitude, robust differences between groups were found. In line with our hypothesis, alcohol-intoxicated individuals displayed reduced No-Go P300 amplitudes. However, Go P300 showed robust differences between groups as well, which is consistent with prior research (Easdon et al. 2005). This suggests that alcohol intake does not specifically impair inhibitory processes as reflected in the No-Go P300, but also results in other executive dysfunctions. In view of the relationship between attentional resource allocation and ERPs, our finding of the larger No-Go N200 and smaller Go and No-Go P300 amplitudes in the alcohol group may hypothetically indicate that the early stage frontal processing occupies more cognitive resources and then leads to a resource depletion in the late-stage inhibitory and execution process. This, in turn, could partly account for the increase in error rate after alcohol intake.

Most interestingly, a significant alcohol × emotion interaction was found. Specifically, the alcohol and placebo participants only differed on angry and happy facial expression trials; no P300 amplitude between-group difference could be observed in response to neutral facial expressions. Furthermore, whereas P300 amplitudes were significantly larger for emotional facial expressions as compared to neutral faces in the placebo group, P300 amplitudes in the alcohol group did not differ between the different facial expressions. In contrast with studies showing that alcohol only influences the processing of positive emotional information (e.g., Kano et al. 2003) or only selectively attenuates negative affect or the processing of negative stimuli (Bartholow et al. 2011; Curtin et al. 2001; Franken et al. 2007; Stevens et al. 2008), our study thus indicates that alcohol consumption reduced the processing of both happy and angry emotional facial expression, suggesting that acute alcohol intake affects emotional processing in general rather than a specific emotion. These results further indicate that in sober controls, emotional expressions facilitated the detection of targets (Go) among the neutral distracter faces (No-Go), while emotional information may have interrupted suppressing and executing responses in alcohol-intoxicated individuals by restricting attentional resources for cognitive control, as instantiated by reduced P300 amplitudes for emotional faces as compared to neutral faces.

An important remark that should be made concerns the negative shift noted in our findings. In comparison with the placebo controls, the overall average wave in the alcohol-intoxicated group showed a broad and relatively long-lasting negative deflection in both Go and No-Go conditions. This negative shift, lasting for the duration of the 800-ms recording period, overlapped temporally with the N200 and P300 amplitude differences distinguishing the alcohol from the control group. Hence, a question relates to the relationship of this observed negative shift to the enhanced N200 and diminished P300 amplitudes of the alcohol-intoxicated participants in our study, as the group differences in No-Go ERPs could also be due to the general cognitive decline after alcohol consumption. By analyzing the difference wave (No-Go minus Go; Nd200), specifying exclusively inhibitory functioning, results revealed that this Nd200 showed significant group main effects: The negative shift did not account for the quite unexpected result of greater N200 in the alcohol group. In contrast, no group differences in the amplitude of the Pd300 were found. Yet, these findings may raise questions about the interpretation of reduced P300 amplitudes frequently seen in studies investigating the effects of acute alcohol consumption. It might be that at least some of those studies have also shown this negative shift, which herein accounts for between-group P300 amplitude differences. Future research should carefully elucidate the extent to which the selective effects on the specific ERP components of interest are separate from the negative shift.

There are a number of other issues that merit consideration when interpreting the results of the current study. One major issue pertains to the interpretation of the results, as behavioral performance in our study was not completely consistent with the ERP results. Though this remains a large difficulty for the broader field, in neurophysiological studies in which alcohol-intoxicated individuals are compared to placebo controls, behavioral data are essential (i.e., increased error rates, reaction time differences) because it is difficult to interpret decreased brain activation versus increased brain activation based on ERP data alone. In our study, while the alcohol group showed reduced P300 amplitudes in Go conditions as compared to controls, omission errors did not show significant differences. In the case of such observations in populations of patients, high-risk participants or alcohol-intoxicated individuals, as in our study, these findings are equivocal. Normal behavioral performance coupled with decreased brain activity may imply increased neural efficiency; however, as the alcohol group in our study did make more commission errors, this explanation is less likely. Another interpretation might be that the behavioral task was not difficult enough to differentiate the performance in the Go conditions between groups, while the ERPs were sensitive enough to show the group effects. Alternatively, it could be that subtle ERP deviations are not yet discernable in behavioral data, though may be of influence during more complex, real-life situations. A second limitation may involve the effect of block switching. Specifically, in Go/No-Go paradigms that comprise stimuli that are targets on some trials and distracters on others, there is often substantial increase in error rate on switch blocks, where the target is different from that of the preceding block (e.g., Derakshan et al. 2009; Eysenck et al. 2007; Rubinstein et al. 2001). Our task also comprised a mix of switch and non-switch blocks, since the order of the six blocks was randomized across subjects. However, we were unable to systematically examine the effect of block switching in our study (i.e., we could not include Block (switch vs. non-switch) as an additional factor in our analysis), and this might have confounded our error data. Further research should counterbalance the serial order of different block types in order to examine whether alcohol affects efficient task-switching performance and whether the demands of task switching would impair the performance even more. Third, our results should be considered in light of our sample. The sample that we recruited was drawn from a young student population with an education level above average and therefore comprises a restricted group. This may limit the generalizability of our findings. Additionally, as only men were included in our study, it remains to be elucidated whether these findings also apply to women. Fourth, we only studied the effect of a moderate alcohol dose, a dose that is relatively low in the context of the levels of alcohol intake typical for the population from which our sample was drawn. Whether higher doses of alcohol would yield similar results might await further research. Fifth, we did not investigate whether responses to emotional facial expressions differed for male and female actors. This may be an interesting topic for further research, since gender of face stimuli has been found to be an important influencing factor on both conscious and more automatic behavioral tendencies (e.g., Erwin et al. 1992; Seidel et al. 2010). Further, it should be noted that the No-Go N200 effect was observed only in the alcohol group, whereas this effect was absent in placebo controls, which is an unexpected finding without a straightforward explanation. However, we did find the theorized No-Go P300 effect, indicating larger P300 amplitudes in response to No-Go trials than Go trials. Hence, when evaluating this component, our paradigm does properly reveal what would be expected. Nevertheless, the lack of a No-Go N200 effect in controls is remarkable and awaits further replication. Finally, it should be born in mind that there may be differences between stimuli that require processing of emotional information (i.e., categorization by emotional content; as was the case in our paradigm) and stimuli that elicit an emotional response. Though it is well known that faces are one of the most salient sources for emotional processing (e.g., Eastwood et al. 2003), it is not clear whether they elicit a robust emotional response. Furthermore, there was no measure of emotional response included in our study. Hence, it could be possible that the results might be different when the latter group of stimuli is used. Further research should address this issue.

Despite these limitations, our study provides important insights into the way alcohol affects the emotional modulation of cognitive control processes. In summary, we show that the behavioral performance deficits in alcohol-intoxicated individuals, particularly in response to angry faces, are accompanied by enhanced No-Go N200 amplitudes (i.e., more negative in voltage), which does not appear to be due to a general cognitive decline, as indexed by the overall negative shift, after alcohol consumption. The early cognitive control processes in alcohol-intoxicated individuals, however, are not modulated by emotional valence. Second, we show that both Go and No-Go P300 amplitudes are significantly diminished after alcohol consumption, but only in response to angry and happy faces. Furthermore, we show that P300 amplitude differentiates emotional from neutral cues in placebo controls, but that alcohol-intoxicated individuals respond in an undifferentiated manner to all facial expressions. In conclusion, our results suggest that alcohol dampens emotional responsiveness, which may restrict the availability of attentional resources for cognitive control. Yet, these findings may underlie the lack of cognitive control frequently seen in alcohol-intoxicated individuals when faced with emotionally or socially challenging situations.
